# Long-Lasting Pathological Mental Fatigue After Brain Injury–A Dysfunction in Glutamate Neurotransmission?

**DOI:** 10.3389/fnbeh.2021.791984

**Published:** 2022-01-31

**Authors:** Lars Rönnbäck, Birgitta Johansson

**Affiliations:** Institute of Neuroscience and Physiology, University of Gothenburg, Gothenburg, Sweden

**Keywords:** brain injury, mental fatigue, astroglia, glutamate transmission, traumatic brain injury, stroke, fatigue, concussion

## Abstract

Long-lasting mental or cognitive fatigue may be a disabling symptom after physically recovered skull trauma, stroke, infection, or inflammation in the central nervous system (CNS). It is difficult to go back to work and participate in familiar social activities, as typically the person is only able to remain mentally active for short periods, and if mentally exhausted, the recovery time will be disproportionally long. Mental fatigue after traumatic brain injury correlates with brain information processing speed. Information processing is energy consuming and requires widespread and specific neural signaling. Glutamate signaling is essential for information processing, including learning and memory. Low levels and the fine-tuning of extracellular glutamate are necessary to maintain a high precision in information processing. The astroglial cells are responsible for the fine-tuning of the glutamate transmission, but this capacity is attenuated by substances or conditions associated with neuro-inflammation in brain pathology. In this paper, we extend our previously presented hypothesis on the cellular mechanisms underlying mental fatigue suggesting a dysfunction in the astroglial support of the glutamate transmission. Changes in other neurotransmitters such as dopamine, serotonin, norepinephrine, GABA, and acetylcholine after brain injury are also taken into consideration.

## Introduction

Fatigue is one of the most common reasons for people to apply for primary care. However, the fatigue is not always visible externally; nor can it be detected or quantified by any blood sample or other test. Fatigue is a private experience. It is difficult to define and to measure in an objective manner. It can be experienced as physical or cognitive/mental fatigue, and may have different neurobiological and neurophysiologic correlates. Fatigue in neurological disorders has been suggested to have a neurobiological origin related to neural circuits that connect the basal ganglia, amygdala, thalamus, and frontal cortex (Chaudhuri and Behan, 2000, 2004). In support of this hypothesis, a dopamine imbalance hypothesis has been presented based on disruption of communication between these regions innervated by dopamine neurons. The dopamine imbalance is shown with structural and functional neuroimaging studies with abnormalities in the frontal and striatal regions (Dobryakova et al., 2015).

In this paper, we focus on pathological mental, or cognitive, fatigue that may be long-lasting after a brain trauma or disease in the nervous system; for symptoms, see Table 1. We extend our previously proposed hypothesis underlying this pathological mental fatigue (Rönnbäck and Hansson, 2004) by suggesting that the brain astroglial cells as a supporting system are dysfunctional concerning the glutamate fine-tuning in glutamate neurotransmission. Such dysfunction could be due to low-grade neuroinflammation together with slight but persistent microglial activation (Hansson and Rönnbäck, 1995; Rönnbäck and Hansson, 2004; Johansson and Rönnbäck, 2014).

**TABLE 1:** Typical symptoms of pathological mental fatigue according to Johansson and Rönnbäck (2014).

• Decreased attention and concentration over time;
• Disproportionally long recovery time after mental exhaustion;
• Subjective memory disturbance;
• Noise sensitivity;
• Light sensitivity;
• Emotional lability;
• Irritability;
• Stress sensitivity;
• Impaired simultaneous capacity;
• Sleep disturbance;
• Headache after mental activity.

## Brain Cell Organization, Glutamate Neurotransmission, and Energy Supply

In consideration of brain cell organization and energy supply, it is estimated that the human brain consists of 10^11^ neurons and 3–5 times as many glial cells. A single neuron may thus have contacts with many thousands of other neurons. Each of these has further synaptic contact points that include a wrapping by astroglial end feet. Several thousands of neuron synapses are connected *via* the end feet of associated astroglial cells. Astroglial cells also have direct end foot-to-end foot connections forming large cell networks (Hansson and Rönnbäck, 2003; Siracusa et al., 2019).

Glutamate is the most important excitatory neurotransmitter in the CNS. It is involved in mental activities such as learning and memory (Niciu et al., 2012). The astroglia regulate the extracellular glutamate levels ([Glu]_*ec*_) and, after a presynaptic release and interaction with the post-synaptic glutamate receptors they clear the extracellular space from excessive glutamate (Hertz and Zielke, 2004). Glutamic acid [Glu]_*ec*_ has to be maintained at approximately 1–3 μM in order to maintain a sufficient signal-to-noise ratio in glutamate transmission (Yudkoff et al., 1993) and to minimize the risk for excitotoxic actions of glutamate on neurons (Choi, 1992). The astrocytes express high-affinity Na^+^-dependent glutamate transporters, the glutamate aspartate transporter (GLAST) and the glutamate transporter 1 (GLT-1) (Danbolt, 2001), prominently located on astrocyte processes surrounding the synapses of glutamatergic neurons (Danbolt, 2001). GLT-1 is considered the most important transporter for removal and regulation of [Glu]_*ec*_ at synaptic sites. GLT-1 is expressed on astrocytes in the presence of glutamatergic neurons (Perego et al., 2000; Niederberger et al., 2003; Björklund et al., 2010).

The human brain requires continuous delivery of glucose and consumes approximately 20% of the total energy budget (Mink et al., 1981). Glucose is the main source of energy for brain functions, including ATP production, oxidative stress management, and synthesis of neurotransmitters, neuromodulators, and structural components (Mink et al., 1981; Mergenthaler et al., 2013). Inadequate energy resources give rise to pathological brain function (Harris et al., 2012; Mergenthaler et al., 2013). The blood-brain barrier (BBB) is selectively permeable for glucose. The concentration gradient drives glucose across the endothelial membrane into the extracellular space *via* GLUT1 membrane transporter. This then mediates glucose uptake into astrocytes, oligodendroglia and microglia. Analogously, GLUT3 facilitates neuronal glucose uptake. Under normal physiological conditions, these cellular glucose uptake rates into neurons are controlled by brain activation (Mergenthaler et al., 2013).

Synaptic transmission needs a constant supply of energy (Mink et al., 1981) and therefore glucose metabolism is critical for information transfer and processing in the brain. Positron emission tomography (FDG-PET) with [(18)F]fluorodeoxyglucose has been used to measure glucose uptake and metabolism in the brain (Byrnes et al., 2014). Astrocytes get energy mostly *via* glycolysis, while for neurons this is primarily by mitochondrial oxidative metabolism. Energy utilization in neurons is related to the activity of ion pumps for establishing electrical gradients which is important for efficient neuronal activation and information transfer. Astrocytes are thus key cells for the coupling between synaptic activity and energy metabolism transfer of lactate to neurons (Magistretti and Allaman, 2015). Both astrocytes and neurons are metabolically upregulated in response to increased neurotransmission (Harris et al., 2012). Glucose is used both as an energy source for the brain and is also a precursor for neurotransmitters (e.g., acetylcholine, glutamate, GABA) as well as neuromodulators (Dienel, 2019).

## Astrogial–Neuronal Interaction During Glutamate Transmission

**FIGURE 1:**
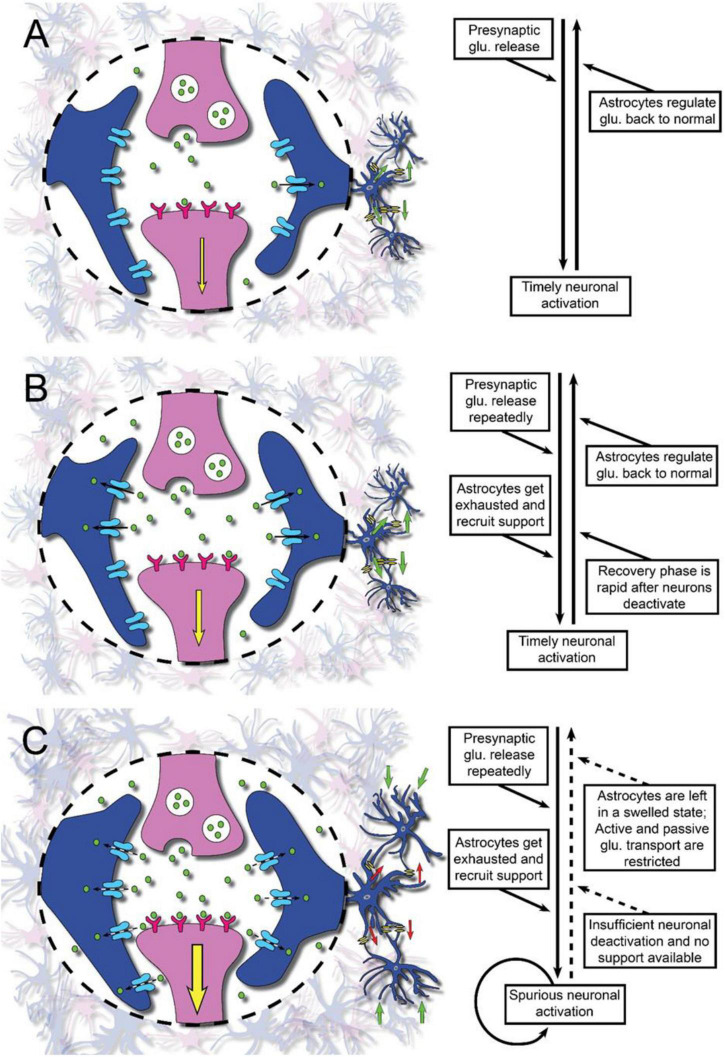
Illustration of glutamate neurotransmission in physiology and the pathophysiology in brain injury or disease as a proposed underlying mechanism of mental fatigue. Synapses with the pre- and post-synaptic membranes (pink) and surrounding astroglial processes (blue) are shown. After interaction with the receptors on the post-synaptic membrane, glutamate (glu.) is removed from the synaptic area by the astroglial cell processes. Panel (A) represents the normal situation with sufficient astroglial support of the neuronal glutamate transmission. Panel (B) represents the situation where there is–still under normal conditions with sufficient astroglial support–a heavy information intake and information processing. The astroglial networks need help from networks located nearby. Panel (C) represents the situation after a traumatic brain injury (TBI) or stroke when there is an attenuated neuronal support by the astroglial networks. Larger parts of the brain than normal will be activated upon mental stimuli. When there is a low flow of information intake and information handling the astroglial support is sufficient for the glutamate transmission. However, if the information to be handled increases, the astroglial support will be insufficient. The astroglial networks are overloaded and the cell networks will increase in volume with a resulting decrease of the extracellular space. If information intake continues, the glutamate signaling will decrease.

Neurons form large networks and signal from and to other neurons. A significant number of synapses could be activated in a mental or cognitive process. The pre- and post-synaptic membranes are encapsulated by astroglial processes, which express the glutamate uptake carriers. The astroglial cells thereby regulate glutamate and ion levels in the synaptic cleft (Ruff et al., 1994; Hansson and Rönnbäck, 1995; Hertz and Zielke, 2004; Perea et al., 2009; Lalo et al., 2021; Semyanov and Verkhratsky, 2021). Upon uptake of glutamate the astroglial cell volume increase somewhat, primarily due to osmosis (Hansson and Rönnbäck, 1995), but will rapidly be restored (Figure 1A). Upon intense glutamate signaling, the astrocytes can recruit help from other astrocytes within the astroglial network (Figure 1B). If there is a prominent glutamate transmission due to activation of large numbers of neurons, the astroglial support may be limited. Intense neuronal activity for a longer period of time could thus result in a saturation of the astroglial support (Figures 1B,C). Furthermore, the transport capacity of extracellular substances will be limited due to the decreased extracellular space (volume transmission) (Sykóva, 2001). It has been shown that glutamate causes astroglial swelling through interaction with metabotropic glutamate receptor 5 (Shi et al., 2017). Even elevated extracellular K^+^ ([K^+^]_*ec*_) induces astroglial swelling (Walch et al., 2020) thus decreasing the extracellular volume. As a consequence, the extracellular glutamate concentration will increase with a resulting triggering of action potentials and the level of mental precision will probably decrease. After a short break, however, the astroglial support of the glutamate transmission will be restored and we are mentally ready for further activities or to resume the mental work we were previously engaged in.

## Energy Crisis and Neurotransmission in Brain Injury and Disease

Information of neuronal activation can be evaluated with FDG-PET, functional magnetic resonance imaging (fMRI), and functional near-infrared spectroscopy (fNIRS) (Magistretti and Allaman, 2015). Sustained hypometabolism or depressed cerebral glucose uptake has been observed in some brain regions after a traumatic brain injury (TBI) (Byrnes et al., 2014), and it was concluded that sustained brain hypometabolism or depressed cerebral uptake of FDG can last for days up to months after mild TBI (mTBI). Hypometabolism was detected after mild/moderate TBI, primarily in the frontal and temporal cortex (Ruff et al., 1994). Decreased glucose uptake was detected in bilateral frontal areas and an increased glucose uptake around the limbic system after mTBI (Komura et al., 2019). To date, there have been no studies relating glucose metabolism to fatigue in humans. However, the relation between hypometabolism and attention has been reported after TBI (Humayun et al., 1989; Gross et al., 1996; Mendeza et al., 2013). Reduced glucose metabolism using FDG-PET has also been found in other neurological patient groups. Glucose metabolism was significantly lower in Multiple Sclerosis (MS) compared to healthy controls, with predominant reduction bilaterally in prefrontal areas and in adjacent white matter. Fatigue was suggested to be related with dysfunctions in frontal cortex and basal ganglia (Roelcke et al., 1997). Those with Parkinson’s disease also suffering from fatigue showed metabolic changes in cortical regions, with lower glucose metabolism in the right insula (Cho et al., 2017), higher metabolism in right middle temporal gyrus and left middle occipital gyrus and lower metabolism in right precuneus and left inferior and superior frontal gyrus (Zhang et al., 2018).

Dysfunction in brain activity within the cortico-striatal-thalamic circuits measured with fMRI is suggested to be related to fatigue after TBI (Kohl et al., 2009; Engström Nordin et al., 2016; Möller et al., 2017; Wylie et al., 2017; Berginström et al., 2018; Skau et al., 2019). With use of fMRI during the performance of a processing speed task, increased brain activity was reported during the test period for people who had suffered a moderate to severe TBI, whereas decreased activity was found for the healthy controls in several brain regions (Kohl et al., 2009). From a study using fNIRS with participants suffering from metal fatigue after mTBI, it was suggested that, compared to healthy controls the mTBI group had a reduced neuronal activity in the frontal cortex (Skau et al., 2019). Reduced brain activity measured with fMRI was found in the basal ganglia, mainly in the caudate nucleus, thalamus, and anterior insula for the TBI group compared to controls. Brain activity diminished during the 27-min test session for the controls whereas the TBI group had a lower activity level, below the controls throughout the whole test session (Berginström et al., 2018). The brain activation (fMRI) in caudate nucleus was reported to be associated to fatigue when comparing a group of TBI participants in relation to healthy controls (Wylie et al., 2017). Abnormal functional connectivity in the thalamus and middle frontal cortex correlated with fatigue in a group of people who had suffered a mTBI (Engström Nordin et al., 2016), as well as altered cerebral blood flow (Möller et al., 2017). Furthermore, a neuroimaging study using fMRI relating to a processing speed task was performed on patients suffering from MS (Chen et al., 2020), a patient group where fatigue is highly prevalent (Shah, 2009). This study showed that the MS patients allocated a lower neuronal activity when exposed to demanding cognitive tasks in comparison with healthy controls (Chen et al., 2020).

Although much is still unknown about glucose metabolism after TBI and brain disorders and diseases and it appears that the hypometabolism and reduced glucose availability in many brain regions could account for the reduced dopamine, noradrenaline, serotonin and acetylcholine signaling and attenuate astrocyte activation with a resulting reduction in glutamate uptake (McGuire et al., 2019). This hypometabolism could account for the emotional symptoms, which can be relieved by low-dose anti-depressive drugs. Further, the attenuated capacity to concentrate can be relieved by, e.g., methylphenidate which increases dopamine and noradrenalin in the synaptic space in frontal brain regions (Arnsten and Dudley, 2005). At least in some states involving disrupted integrity of the BBB, the glucose uptake into the nervous system would appear to be attenuated (Patching, 2017; Noe et al., 2020).

## Astroglial Dysfunction With Impaired Glutamate Uptake and Mental Fatigue–A Hypothesis

If the astroglial fine-tuning of [Glu]_*ec*_ is impaired, this would result in decreased precision in the glutamate signaling (Hansson and Rönnbäck, 1995; Hertz and Zielke, 2004; Rönnbäck and Hansson, 2004; Johansson and Rönnbäck, 2014). The signals taken into the brain and processed there will then be less distinct and probably more unspecific. More information-bearing signals will be recognized as “new” by sensory centers in the brain, and thus reach the cerebral cortex for further handling. Thereby, larger neuronal circuits would be activated. The impaired [Glu]_*ec*_ clearing from the synaptic space may cause local [Glu]_*ec*_ to increase which could give rise to astrocyte swelling with the concomitant shrinking of the extracellular space (Sykóva, 2001). Due to a decrease in extracellular space, diffusion of [Glu]_*ec*_ to adjacent neurons could activate nearby neurons in a non-specific way. In a learning and adaptation process, this will result in spatially altered pathways. As a result, many more synapses will be activated and even more astrocytes must be activated to support this increased neuronal activity. If there are several other processes with similar problems at the same time, the astroglial networks will not manage to maintain their tasks. As a result, an even more extended cell swelling will develop and there will be significant disturbances in sensitive ion transport *via* the extracellular space, i.e., decreased volume transmission (Figure 1C). Upon astroglial volume increase the cell membranes will depolarize slightly, which will further impair astroglial glutamate uptake, and also impair the astroglial capacity to remove [K^+^]_*ec*_. Even slightly elevated [K^+^]_*ec*_ levels, up to 8–10 mM, have been shown to diminish glutamate release from the presynaptic terminals (Meeks and Mennerick, 2004). Astroglial cell volume is also known to increase when [K^+^]_*ec*_ increases (Walch et al., 2020). In addition, it has been shown that when astroglial glutamate uptake is impaired, even the glucose uptake by the astrocytes decreases (Hertz and Zielke, 2004) and consequently the supply of metabolic substrates to the neurons. In addition, decreased glutamine supply to the neurons due to the impaired astroglial handling of glutamate will result in a metabolic depletion concerning the glutamate transmission and thereby decreased glutamate release from the presynaptic terminals. Decrease in glucose supply and thereby attenuation of glutamate transmission will also reduce GABA formation and transmission. This is especially interesting as studies using fMRI from the caudate nucleus have demonstrated impaired glucose metabolism (see above and Wylie et al., 2017). In fact, in the caudate nucleus more than 90% of the cells are GABAergic. Fast-spiking GABAergic neurons represent a prominent number of feed-forward inhibition in many cortical and subcortical regions and fire at a high rate. They could also exhaust their transmitter pool and further contribute to the glutamate pathology. GABA has been shown to be involved in cognitive impairments (Ghosal et al., 2017; Cao et al., 2018) and might be of importance for information filtering in the brain (Hammett et al., 2020), which are symptoms of importance in mental/cognitive fatigue.

Thus, our hypothesis could explain why persons with these mental fatigue symptoms might be able to perform cognitive tasks just for short periods. However, in situations with heavy sensory stimulation, they will feel exhausted and it will take unproportionally long time to recover. They may be defined as having a Brain Energy Deficiency Disorder (BEDD).

## How Can the Mental Fatigue Become Long-Lasting Even After the Brain Trauma or Disease has Healed?

It has been shown that overstimulation of the astrocyte networks, for instance in a state of neuroinflammation will lead to extracellular ATP signaling between the astroglial cells (Rodrigues et al., 2015). This will result in ATP interaction with microglial cells (Inoue, 2002) and even with brain mast cells (Traina, 2019) or pathological pericyte activation in the blood cell wall (Kawamura et al., 2003) thereby continuing the neuroinflammation and also the BBB disruption (Erickson et al., 2012; Cervellati et al., 2020). A vicious circle is created which can be difficult to stop once activated. Furthermore, in persons with premorbid inflammatory activity either within the CNS or outside it, the likelihood of being able to stop the injury or disease-induced neuroinflammation with disturbed BBB function may be limited.

## Support for Our Hypothesis

It is well-known that TBI and ischemia as well as degenerative disorders are associated with neuroinflammation with disturbed integration of the BBB (Abbott et al., 2006; Profaci et al., 2020). Similarly mental/cognitive fatigue is common in autoimmune diseases such as MS, hypo- or hyperthyreosis and rheumatoid arthritis where there is an obvious neuroinflammation. It is interesting to note that in states of disease or disorder in the nervous system BBB integrity is affected (Abbott et al., 2006) and BBB seems more vulnerable to systemic inflammation (Varatharaj and Galea, 2017) thus with the possibility to maintain the neuroinflammation over time.

Cell swelling is another part of the hypothesis. The mechanisms behind cell volume variations are relatively well-understood, but their role in brain function remains largely unexplored. Osmosis is an essential driving force. Several astroglial processes change intracellular ion concentrations, which cause the cell to swell or shrink. An increase in volume is normally reversed as metabolized substances are released. Once we have a state with easily super-expandable cells, we will readily reach a state of limited extra cellular space. This, in turn could lead us to a result in terms of the perception of mental fatigue. Even [Glu]_*ec*_, as mentioned above, mediates astroglial volume increase (Shi et al., 2017), as well as elevated [K^+^]_*ec*_ (Walch et al., 2020).

It is well-known that persons who suffer from pathological fatigue with impaired attention and concentration over time often have additional, quite different symptoms as well. These may include a more generalized physical fatigue, long-term pain, and autonomic nervous system symptoms. A regulatory imbalance across the hypothalamus-hypophysis-adrenal cortex (HPA)-axis, which is under the control of glutamate has been demonstrated (Mathew et al., 2001). It has also been shown that glutamate can activate the amygdala to release corticotropine-releasing factor (CRF) (Cratty and Birkle, 1999), which, in turn can induce increased levels of glucocorticoids. In the long-term increased levels of glucocorticoids have been shown to interfere with glutamate transmission (Popoli et al., 2011). In this respect, it is of interest to mention that acute stimulation of CRF will result in increased attention as well as stimulation of the hypothalamic production of β-endorphins and pain relief. However, on the other hand, a long-term activation, with a down-regulation of the HPA-axis could result in fatigue, depressive mood, increased pain sensitivity and also increased sensitivity to infections and inflammatory states (Tsigos and Chrousos, 2002).

## Aspects on Further Research

Some different approaches could be directed toward the following:

• Brain imaging on humans suffering from mental fatigue – following extracellular concentrations of Glu, K^+^, ATP, and neurotropic/neuroactive substances over time;
• Clinical studies with different treatment strategies;
• Animal experiments – role of astroglial network and defect glutamate uptake;
• *In vitro* experiments–effects of specific substances on glial-glial/glial-neuronal signaling and glial properties;
• More knowledge about the roles and mechanisms of pericytes as regulators of BBB function and stability.

## Data Availability Statement

The original contributions presented in the study are included in the article/supplementary material, further inquiries can be directed to the corresponding author.

## Author Contributions

LR extended the previously presented hypothesis and wrote this manuscript in cooperation with BJ. Both authors contributed to the article and approved the submitted version.

## Conflict of Interest

The authors declare that the research was conducted in the absence of any commercial or financial relationships that could be construed as a potential conflict of interest.

## Publisher’s Note

All claims expressed in this article are solely those of the authors and do not necessarily represent those of their affiliated organizations, or those of the publisher, the editors and the reviewers. Any product that may be evaluated in this article, or claim that may be made by its manufacturer, is not guaranteed or endorsed by the publisher.

## References

[B1] Abbott N. J. Rönnbäck L. Hansson E. (2006). Astrocyte-endothelial interactions at the blood-brain barrier. *Nat. Rev. Neurosci.* 7 41–53. 10.1038/nrn1824 16371949

[B2] Arnsten A. F. T. Dudley A. G. (2005). Methylphenidate improves prefrontal cortical cognitive function through α2 adrenoceptor and dopamine D1 receptor actions: relevance to therapeutic effects in attention deficit hyperactivity disorder. *Behav. Brain Funct.* 1:2. 10.1186/1744-9081-1-2 15916700 PMC1143775

[B3] Berginström N. Nordström P. Ekman U. Eriksson J. Andersson M. Nyberg L. (2018). Using functional magnetic resonance imaging to detect chronic fatigue in patients with previous traumatic brain injury: changes linked to altered striato-thalamic-cortical functioning. *J. Head Trauma Rehabil.* 33 266–274. 10.1097/HTR.0000000000000340 28926483

[B4] Björklund U. Persson M. Rönnbäck L. Hansson E. (2010). Primary cultures from cerebral cortex and hippocampus enriched in glutamatergic and gabaergic neurons. *Neurochem. Res.* 35 1733–1742. 10.1007/s11064-010-0236-x 20680458

[B5] Byrnes K. R. Wilson C. M. Brabazon F. von Leden R. Jurgens J. S. Oakes T. R. (2014). FDG-PET imaging in mild traumatic brain injury: a critical review. *Front. Neuroenerget.* 5:13. 10.3389/fnene.2013.00013 24409143 PMC3885820

[B6] Cao G. Edden R. A. E. Gao F. Li H. Gong T. Chen W. (2018). Reduced GABA levels correlate with cognitive impairment in patients with relapsing-remitting multiple sclerosis. *Eur. Radiol.* 28 1140–1148. 10.1007/s00330-017-5064-9 28986640 PMC5812783

[B7] Cervellati C. Trentini A. Pecorelli A. Valacchi G. (2020). Inflammation in neurological disorders: the thin boundary between brain and periphery. *Antioxid. Redox Signal.* 33 191–210. 10.1089/ars.2020.8076 32143546

[B8] Chaudhuri A. Behan P. O. (2000). Fatigue and basal ganglia. *J. Neurol. Sci.* 179 34–42. 11054483 10.1016/s0022-510x(00)00411-1

[B9] Chaudhuri A. Behan P. O. (2004). Fatigue in neurological disorders. *Lancet* 363 978–988. 15043967 10.1016/S0140-6736(04)15794-2

[B10] Chen M. H. Wylie G. R. Sandroff B. M. Dacosta-Aguayo R. DeLuca J. Genova H. M. (2020). Neural mechanisms underlying state mental fatigue in multiple sclerosis: a pilot study. *J. Neurol.* 267 2372–2382. 10.1007/s00415-020-09853-w 32350648

[B11] Cho S. S. Aminian K. Li C. Lang A. E. Houle S. Strafella A. P. (2017). Fatigue in Parkinson’s disease: the contribution of cerebral metabolic changes. *Hum. Brain Mapp.* 38 283–292. 10.1002/hbm.23360 27571419 PMC6867035

[B12] Choi D. W. (1992). Excitotoxic cell death. *J. Neurobiol.* 23 1261–1276. 1361523 10.1002/neu.480230915

[B13] Cratty M. S. Birkle D. L. (1999). N-methyl-D-aspartate (n.d.)-mediated corticotropin-releasing factor (CRF) release in cultured rat amygdala neurons. *Peptides* 20 93–100. 10.1016/s0196-9781(98)00147-8 10098629

[B14] Danbolt N. C. (2001). Glutamate uptake. *Prog. Neurobiol.* 65 1–105. 11369436 10.1016/s0301-0082(00)00067-8

[B15] Dienel G. A. (2019). Brain glucose metabolism: integration of energetics with function. *Physiol. Rev.* 1 949–1045. 10.1152/physrev.00062.2017 30565508

[B16] Dobryakova E. Genova H. M. DeLuca J. Wylie G. R. (2015). The dopamine imbalance hypothesis of fatigue in multiple sclerosis and other neurological disorders. *Front. Neurol.* 12:52. 10.3389/fneur.2015.0005 PMC4357260 25814977

[B17] Engström Nordin L. Möller M. C. Julin P. Bartfai A. Hashim F. Li T.-Q. (2016). Post mTBI fatigue is associated with abnormal brain functional connectivity. *Sci. Rep.* 6:21183. 10.1038/srep21183 26878885 PMC4754765

[B18] Erickson M. A. Dohi K. Banks W. A. (2012). Neuroinflammation: a common pathway in CNS diseases as mediated at the blood-brain barrier. *Neuroimmunomodulation* 19 121–130. 10.1159/000330247 22248728 PMC3707010

[B19] Ghosal S. Hare B. Duman R. S. (2017). Prefrontal cortex GABAergic deficits and circuit dysfunction in the pathophysiology and treatment of chronic stress and depression. *Curr. Opin. Behav. Sci.* 14 1–8. 10.1016/j.cobeha.2016.09.012 27812532 PMC5086803

[B20] Gross H. Kling A. Henry G. Herndon C. Lavretsky H. (1996). Local cerebral glucose metabolism in patients with long-term behavioral and cognitive deficits following mild traumatic brain injury. *J. Neuropsychiatry Clin. Neurosci.* 8 324–334. 10.1176/jnp.8.3.324 8854305

[B21] Hammett S. T. Cook E. Hassan O. Hughes C.-A. Rooslien H. Tizkar R. (2020). GABA, noise and gain in human visual cortex. *Neurosci. Lett.* 736:135294. 10.1016/j.neulet.2020.135294 32777347 PMC7511597

[B22] Hansson E. Rönnbäck L. (1995). Astrocytes in glutamate neurotransmission. Receptor-mediated regulation of uptake carriers, ion channels and cell volume. *FASEB J.* 9 343–350. 7534736 10.1096/fasebj.9.5.7534736

[B23] Hansson E. Rönnbäck L. (2003). Glial neuronal signaling in the central nervous system. *FASEB J.* 17 431–438. 10.1096/fj.02-0429rev 12631574

[B24] Harris J. J. Jolivet R. Attwell D. (2012). Synaptic energy use and supply. *Neuron Rev.* 75 762–777. 10.1016/j.neuron.2012.08.019 22958818

[B25] Hertz L. Zielke H. R. (2004). Astrocytic control of glutamatergic activity: astrocytes as stars of the show. *Trends Neurosci.* 27 735–743. 10.1016/j.tins.2004.10.008 15541514

[B26] Humayun M. S. Presty S. K. Lafrance N. D. Holcomb H. H. Loats H. Long D. M. (1989). Local cerebral glucose abnormalities in mild closed head injured patients with cognitive impairments. *Nucl. Med. Commun.* 10 335–344. 10.1097/00006231-198905000-00004 2787008

[B27] Inoue K. (2002). Microglial activation by purines and pyrimidines. *Glia* 40 156–163. 10.1002/glia.10150 12379903

[B28] Johansson B. Rönnbäck L. (2014). “Long-lasting mental fatigue after traumatic brain injury – a major problem most often neglected diagnostic criteria, assessment, relation to emotional and cognitive problems, cellular background, and aspects on treatment,” in *Traumatic Brain Injury*, ed. Sadaka F. (Rijeka: INTECH).

[B29] Kawamura H. Sugiyama T. Wu D. M. Kobayash I. M. Yamanishi S. Katsumura K. (2003). ATP: a vasoactive signal in the pericyte-containing microvasculature of the rat retina. *J. Physiol.* 15(551(Pt 3)) 787–799. 10.1113/jphysiol.2003.047977 12876212 PMC2343299

[B30] Kohl A. D. Wylie G. R. Genova H. M. Hillary F. G. DeLuca J. (2009). The neural correlates of cognitive fatigue in traumatic brain injury using functional MRI. *Brain Inj.* 23 420–432. 10.1080/02699050902788519 19408165

[B31] Komura A. Kawasaki T. Yamada Y. Uzuyama S. Asano Y. Shinoda J. (2019). Cerebral glucose metabolism in patients with chronic mental and cognitive sequelae after a single blunt mild traumatic brain injury without visible brain lesions. *J. Neurotrauma* 36 641–649. 10.1089/neu.2018.5641 29921156

[B32] Lalo U. Koh W. Lee C. J. Pankratov Y. (2021). The tripartite glutamatergic synapse. *Neuropharmacology* 199:108758. 10.1016/j.neuropharm.2021.108758 34433089

[B33] Magistretti P. J. Allaman I. (2015). A cellular perspective on brain energy metabolism and fuctional imaging. *Neuron Rev.* 86 883–901. 10.1016/j.neuron.2015.03.035 25996133

[B34] Mathew S. J. Coplan J. D. Schoepp D. D. Smith E. L. Rosenblum L. A. Gorman J. M. (2001). Glutamate-hypothalamic-pituitary-adrenal axis interactions: implications for mood and anxiety disorders. *CNS Spect.* 6 561–564. 10.1017/s1092852900002091 15573019

[B35] McGuire J. L. Ngwenya L. B. Robert E. McCullumsmith R. E. (2019). Neurotransmitter changes after traumatic brain injury: an update for new treatment strategies. *Mol. Psychiatry* 24 995–1012. 10.1038/s41380-018-0239-6 30214042

[B36] Meeks J. P. Mennerick S. (2004). Selective effects of potassium elevations on glutamate signaling and action potential conduction in hippocampus. *J. Neurosci.* 24 197–206. 10.1523/JNEUROSCI.4845-03.2004 14715952 PMC6729587

[B37] Mendeza M. F. Owensa E. O. Berenjid G. R. Pepperse D. C. Liangf L.-J. Lichtc E. A. (2013). Mild traumatic brain injury from primary blast vs. blunt forces: post-concussion consequences and functional neuroimaging. *Neurorehabilitation* 32 397–407. 10.3233/NRE-130861 23535805

[B38] Mergenthaler P. Lindauer U. Dienel G. A. Meisel A. (2013). Sugar for the brain: the role of glucose in physiological and pathological brain function. *Trends Neurosci.* 36 587–597. 10.1016/j.tins.2013.07.001 23968694 PMC3900881

[B39] Mink J. W. Blumenschine R. J. Adams D. B. (1981). Ratio of central nervous system to body metabolism in vertebrates: its constancy and functional basis. *Am. J. Physiol.* 421 R203–R210. 10.1152/ajpregu.1981.241.3.R203 7282965

[B40] Möller M. C. Nordin L. E. Bartfai A. Julin P. Li T.-Q. (2017). Fatigue and cognitive fatigability in mild traumatic brain injury are correlated with altered neural activity during vigilance test performance. *Front. Neurol.* 21:496. 10.3389/fneur.2017.00496 28983280 PMC5613211

[B41] Niciu M. J. Kelmendi B. Sanacora G. (2012). Overview of glutamatergic neurotransmission in the nervous system. *Pharmacol. Biochem. Behav.* 100 656–664. 10.1016/j.pbb.2011.08.008 21889952 PMC3253893

[B42] Niederberger E. Schmidtko A. Rothstein J. D. Geisslinger G. Tegeder I. (2003). Modulation of spinal nociceptive processing through the glutamate transporter GLT-1. *Neuroscience* 116 81–87. 10.1016/s0306-4522(02)00547-x 12535941

[B43] Noe C. R. Noe-Letschnig M. Handschuh P. Noe C. A. Lanzenberger R. (2020). Dysfunction of the blood-brain barrier—a key step in neurodegeneration and dementia. *Aging Neurosci.* 12:185. 10.3389/fnagi.2020.00185 32848697 PMC7396716

[B44] Patching S. G. (2017). Glucose transporters at the blood-brain barrier: function, regulation and gateways for drug delivery. *Mol. Neurobiol.* 54 1046–1077. 10.1007/s12035-015-9672-6 26801191

[B45] Perea G. Navarrete M. Araque A. (2009). Tripartite synapses: astrocytes process and control synaptic information. *Trends Neurosci.* 32 421–431. 10.1016/j.tins.2009.05.001 19615761

[B46] Perego C. Vanoni C. Boss I. M. Massari S. Basudev H. Longhi R. (2000). The GLT-1 and GLAST glutamate transporters are expressed on morphologically distinct astrocytes and regulated by neuronal activity in primary hippocampal cocultures. *J. Neurochem.* 75 1076–1084. 10.1046/j.1471-4159.2000.0751076.x 10936189

[B47] Popoli M. Yan Z. McEwen B. Sanacora G. (2011). The stressed synapse: the impact of stress and glucocorticoids on glutamate transmission. *Nat. Rev. Neurosci.* 13 22–37. 10.1038/nrn3138 22127301 PMC3645314

[B48] Profaci C. P. Munji R. N. Pulido R. S. (2020). Daneman R. The blood–brain barrier in health and disease: important unanswered questions. *J. Exp. Med.* 217:e20190062. 10.1084/jem.20190062 32211826 PMC7144528

[B49] Rodrigues R. J. Tomé A. R. Cunha R. A. (2015). ATP as a multi-target danger signal in the brain. *Front. Neurosci.* 9:148. 10.3389/fnins.2015.00148 25972780 PMC4412015

[B50] Roelcke U. Kappos L. Lechner-Scott J. Brunnschweiler H. Huber S. Ammann W. (1997). Reduced glucose metabolism in the frontal cortex and basal ganglia of multiple sclerosis patients with fatigue: a 18f-fluorodeoxyglucose positron emission tomography study. *Neurology* 48 1566–1571. 10.1212/wnl.48.6.1566 9191767

[B51] Rönnbäck L. Hansson E. (2004). On the potential role of glutamate transport in mental fatigue. *J. Neuroinflamm.* 1:22. 10.1186/1742-2094-1-22 15527505 PMC533886

[B52] Ruff R. M. Crouch J. A. Tröster A. I. Marshall L. F. Buchsbaum M. S. Lottenberg S. (1994). Selected cases of poor outcome following a minor brain trauma: comparing neuropsychological and positron emission tomography assessment. *Brain Inj.* 8 297–308. 10.3109/02699059409150981 8081345

[B53] Semyanov A. Verkhratsky A. (2021). Astrocytic processes: from tripartite synapses to the active milieu. *Trends Neurosci.* 44 781–792. 10.1016/j.tins.2021.07.006 34479758

[B54] Shah A. (2009). Fatigue in multiple sclerosis. *Phys. Med. Rehabil. Clin. N. Am.* 20 363–372. 19389617 10.1016/j.pmr.2008.12.003

[B55] Shi Z. Zhang W. Lu Y. Lu Y. Xu L. Fang Q. (2017). Aquaporin 4-mediated glutamate-induced astrocyte swelling is partially mediated through metabotropic glutamate receptor 5 activation. *Front. Cell Neurosci.* 11:116. 10.3389/fncel.2017.00116/full 28503134 PMC5408017

[B56] Siracusa R. Fusco R. Cuzzocrea S. L. (2019). Astrocytes: role and functions in brain pathologies. *Front. Pharmacol.* 10:1114. 10.3389/fphar.2019.01114 31611796 PMC6777416

[B57] Skau S. Bunketorp-Käll L. Kuhn H. G. Johansson B. (2019). Mental fatigue and functional near-infrared spectroscopy (fNIRS) – based assessment of cognitive performance after mild traumatic brain injury. *Front. Hum. Neurosci.* 13:145. 10.3389/fnhum.2019.00145 31139065 PMC6527600

[B58] Sykóva E. (2001). Glial diffusion barriers during aging and pathological states. *Prog. Brain Res.* 132 339–363. 10.1016/S0079-6123(01)32087-3 11545002

[B59] Traina G. (2019). Mast cells in gut and brain and their potential role as an emerging therapeutic target for neural diseases. *Front. Cell Neurosci.* 13:345. 10.3389/fncel.2019.00345 31417365 PMC6682652

[B60] Tsigos C. Chrousos G. P. (2002). Hypothalamic-pituitary-adrenal axis, neuroendocrine factors and stress. *J. Psychosom. Res.* 53 865–871. 10.1016/s0022-3999(02)00429-4 12377295

[B61] Varatharaj A. Galea I. (2017). The blood-brain barrier in systemic inflammation. *Brain Behav. Immun.* 60 1–12. 10.1016/j.bbi.2016.03.010 26995317

[B62] Walch E. Murphy T. R. Cuvelier N. Aldoghmi M. Morozova C. Donohue J. (2020). Astrocyte-selective volume increase in elevated extracellular potassium conditions is mediated by the Na +/K + ATPase and occurs independently of aquaporin 4. *ASN Neuro.* 12:1759091420967152. 33092407 10.1177/1759091420967152 PMC7586494

[B63] Wylie G. R. Dobryakova E. DeLuca J. Chiaravalloti N. Essad K. Genova H. (2017). Cognitive fatigue in individuals with traumatic brain injury is associated with caudate activation. *Sci. Rep.* 7:8973. 10.1038/s41598-41017-08846-41596 28827779 PMC5567054

[B64] Yudkoff M. Nissim I. Daikhin Y. Lin Z. P. Nelson D. Pleasure D. (1993). Brain glutamate metabolism: neuronal-astroglial relationships. *Dev. Neurosci.* 15 343–350. 10.1159/000111354 7805588

[B65] Zhang L. Li T. Yuan Y. Tong Q. Jiang S. Wang Ml (2018). Brain metabolic correlates of fatigue in Parkinson’s disease: a PET study. *Int. J. Neurosci.* 128 330–336. 10.1080/00207454.2017.1381093 28918694

